# COVID-19 Exposure, Protective Measures, Symptom Assessment, and Risk Perception Among Healthcare Workers in Peru: A Longitudinal Cohort Study (2020–2021)

**DOI:** 10.1093/cid/ciaf343

**Published:** 2025-07-29

**Authors:** Diana Ponce, Matthew Westercamp, Giselle Soto, Fernanda C Lessa, Perrine Marcenac, Candice Romero, María Silva, Rachel Smith, Garret Mahon, Alejandro Llanos-Cuentas, Eduardo Matos, Michael Prouty, Andres Espinosa-Bode, Joan Neyra

**Affiliations:** Vysnova Partners, Inc., Alexandria, Virginia, USA assigned to Clinical Trials Unit, NAMRU SOUTH; Division of Healthcare Quality Promotion, U.S. Centers for Disease Control and Prevention (CDC), Atlanta, Georgia, USA; Clinical Trials Unit, U.S. Naval Medical Unit Research SOUTH (NAMRU SOUTH), Lima, Peru; Division of Healthcare Quality Promotion, U.S. Centers for Disease Control and Prevention (CDC), Atlanta, Georgia, USA; Influenza Division, U.S. Centers for Disease Control and Prevention (CDC), Atlanta, Georgia, USA; Vysnova Partners, Inc., Alexandria, Virginia, USA assigned to Clinical Trials Unit, NAMRU SOUTH; Clinical Trials Unit, U.S. Naval Medical Unit Research SOUTH (NAMRU SOUTH), Lima, Peru; Division of Healthcare Quality Promotion, U.S. Centers for Disease Control and Prevention (CDC), Atlanta, Georgia, USA; Division of Healthcare Quality Promotion, U.S. Centers for Disease Control and Prevention (CDC), Atlanta, Georgia, USA; Infectious Diseases and Tropical Medicine Department, Cayetano Heredia Hospital, Lima, Peru; Infectious Diseases Department, Arzobispo Loayza National Hospital, Lima, Peru; Clinical Trials Unit, U.S. Naval Medical Unit Research SOUTH (NAMRU SOUTH), Lima, Peru; Division of Healthcare Quality Promotion, U.S. Centers for Disease Control and Prevention (CDC), Atlanta, Georgia, USA; Clinical Trials Unit, U.S. Naval Medical Unit Research SOUTH (NAMRU SOUTH), Lima, Peru

**Keywords:** COVID-19, healthcare workers, risk perception, protective measures, occupational exposure

## Abstract

**Background:**

The coronavirus disease 2019 (COVID-19) pandemic placed significant pressure on healthcare workers (HCWs) globally, exposing them to high risks of infection and psychological stress. This study assessed dynamic risks and protective factors for severe acute respiratory syndrome coronavirus 2 (SARS-CoV-2) infection among HCWs in Lima, Peru.

**Methods:**

We conducted the Peru Healthcare Worker COVID Cohort (PHCWCC), a prospective cohort study during the pre-COVID-19 vaccination period (August 2020–May 2021) and the post-vaccination period (August 2021–May 2022). Data included weekly nasal swabs for SARS-CoV-2 testing, reports on exposures, symptoms, perceived risks, and vaccination status. Incidence and factors associated with SARS-CoV-2 positivity were analyzed.

**Results:**

Among 1369 HCWs, the infection rate increased from 1.15 (95% confidence interval [CI]: .90–1.14) to 1.71 (95% CI: 1.41–2.05) per 1000 HCW-days between the pre- and post-vaccination phases (*P* = .01). Despite 97% receiving the primary vaccine series and 75% a booster, perceived high infection risk rose from 26% to 35% (*P* < .001). Close contact with infected colleagues remained a consistent risk factor (odds ratio [OR] = 2.74, 95% CI: 1.85–4.05; *P* < .001). In the second phase, direct patient contact (OR = 1.92, 95% CI: 1.20–3.08; *P* = .006) and patient care environments (OR = 2.14, 95% CI: 1.35–3.40; *P* = .001) were linked to higher infection risk. Perceived infection risk was inversely associated with positivity (OR = 0.76, 95% CI: 0.63–0.91; *P* = .003).

**Conclusions:**

Evolving infection risks highlight the need for sustained infection prevention, including during non-patient care activities. Fostering risk awareness may reduce infection rates and strengthen healthcare system resilience.

## Teaser

This study analyzes coronavirus disease 2019 (COVID-19) risk factors and symptoms among healthcare workers in Peru, highlighting occupational risks, the role of protective measures, and the crucial need for ongoing surveillance and adaptive strategies amidst evolving epidemiological challenges.

## BACKGROUND

On 11 March 2020, the World Health Organization (WHO) declared the coronavirus disease 2019 (COVID-19) outbreak a global pandemic [1], triggering an urgent global public health response. This included the development of strategies to mitigate exposure and manage transmission safeguards among populations. Central to these efforts are healthcare workers (HCWs), who are critical in maintaining healthcare systems while facing multiple challenges such as pathogen exposure, physical exhaustion, and psychological burdens, including stigma, fear, and depression [2].

The pandemic exerted unprecedented pressures on healthcare systems worldwide, highlighting the vulnerability of HCWs to infection and their potential role in amplifying disease transmission [3]. South America was particularly affected, with Brazil detecting its first case on 26 February 2020 [4], and Peru confirming its first case in early March [5]. By 1 September 2020, Peru reported the world's highest COVID-19 per capita death rate, with an alarming case-fatality ratio and over 74 000 cumulative deaths [6]. These early months uncovered critical deficiencies in Peru's healthcare infrastructure, including under-resourced facilities, staffing shortages, inadequate diagnostic capacities, and a severe lack of personal protection equipment (PPE), which posed risks to HCWs and hindered effective pandemic response [7–9].

This study evaluates a cohort of HCWs in Lima, Peru, during 2 distinct phases: the pre-COVID-19 vaccination period from August 2020 to May 2021, and the post-COVID-19 vaccination period from August 2021 to May 2022, aligning with the emergence of the Omicron variant in Peru. We provide a detailed analysis of characteristics and occupational risk factors assessed throughout follow-up, along with weekly testing for incident infections and symptom reporting.

Understanding both perceived and actual risk is essential when evaluating HCW vulnerability during public health emergencies. There is well-documented evidence of the psychological burdens and negative mental health impacts associated with prolonged periods of elevated risk perception, especially among HCWs early in the COVID-19 response [10–15]. However, it has also been observed that higher perceived risk can motivate adherence to recommended infection prevention practices, ultimately reducing the actual risk of infection [16–19].

Unlike studies from high-income settings, which have focused on HCWs’ infection risks and mental health in well-resourced healthcare environments, this study provides critical insights into the experiences of HCWs in a low- and middle-income country (LMIC) setting facing more pronounced healthcare system constraints. Maintaining cohorts of HCWs throughout public health emergencies like this one provides a valuable opportunity to explore these dynamics as the response evolves. By examining both perceived and actual risk in an LMIC context, this study contributes to a broader understanding of how resource limitations shape HCW safety and response behaviors, offering comparisons to studies conducted in other regions.

## METHODS

### Setting

The Peru Healthcare Worker COVID Cohort (PHCWCC) is a sub-study that builds on the original Estudio Vacuna de Influenza Peru (VIP), a prospective cohort study of healthcare workers (HCWs) in Lima, Peru, which was established in 2016 and concluded in 2019. During the COVID-19 pandemic, a new cohort with similar characteristics and target population was re-established to continue surveillance activities, expanding beyond influenza to also include SARS-CoV-2. This article focuses on data collected during two key follow-up periods: a pre-COVID-19 vaccination phase (PHCWCC-1) and a subsequent post-COVID-19 vaccination phase approximately 3 months later (PHCWCC-2).

Recruitment for PHCWCC-1 began amid logistical challenges induced by the pandemic, starting at Cayetano Heredia Hospital from August to October 2020 and extending to Archbishop Loayza National Hospital from November 2020 to January 2021. Each participant was monitored over a 16-week period, with follow-up concluding in May 2021.

The recruitment and follow-up structure for PHCWCC-2 mirrored that of the first phase, beginning in August 2021 and concluding in May 2022. Study activities were paused from June to July 2021, to align operational timelines with the parent study. Although this pause was necessary for logistical reasons, it occurred during a dynamic phase of the pandemic, which may have influenced contextual factors between the 2 periods. However, study design, data collection protocols, and participant follow-up methods remained consistent across both phases to support comparability.

### Eligibility Criteria

Eligible participants were HCWs enrolled in VIP, aged 18 years or older, working at least 30 hours per week, with routine direct patient contact (within 1 meter). They were required to have been employed by the hospital for at least 1 year and to have no plans to leave employment in the coming year. Notably, enrollment in PHCWCC-2 did not require prior participation in PHCWCC-1.

### Recruitment Strategy and Enrollment

VIP recruitment employed a stratified sampling strategy, as previously described [20]. Stratification was based on age group (3 categories), sex (2 categories), and occupational group (3 categories) resulting in 18 strata applied across all HCWs at each hospital. The target was to enroll at least 50 participants per stratum.

HCWs were invited to participate through (1) informational meetings at their respective hospital services and (2) direct contact via phone or in person. The overall acceptance rate was 72%.

Upon entering the VIP cohort, participants from designated healthcare facilities were invited to join the PHCWCC. The PHCWCC enrollment process included a survey capturing sociodemographic characteristics, work responsibilities, personal protective equipment availability, health status, health behaviors, and both known and suspected COVID-19 exposures. The survey also assessed knowledge, attitudes, and practices (KAP) regarding COVID-19 and infection risk.

At enrollment, participants provided a self-collected nasal swab for baseline SARS-CoV-2 testing.

### Weekly Follow-up

For a period of 16 weeks, participants completed a weekly COVID-19 cohort-specific questionnaire that included queries about COVID-19 exposures, protective measures taken, and symptoms compatible with COVID-19 not previously reported through active surveillance during the previous week. Regardless of symptom presence, participants provided a self-collected nasal swab each week.

Participants who reported providing care for COVID-19 patients were asked whether they felt able to follow safety and PPE protocols while providing care. This question assessed perceived ability to follow protocols without exploring underlying reasons. Questions about PPE availability were assessed separately.

Healthcare workers’ perception of their risk of acquiring COVID-19 at the hospital was assessed by participants self-rating their perceived risk on a scale from 1 (very low) to 10 (very high) and indicated their overall sense of safety for themselves and their coworkers.

Vaccination for HCWs at participating facilities commenced on 9 February 2021 [21], with primary series vaccination continuing beyond the PHCWCC-1 follow-up period. Initially, all eligible HCWs were offered the Sinopharm BBIBP-CorV (Beijing Institute of Biological Products Coronavirus Vaccine) inactivated whole virus vaccine, with the 2 recommended doses administered 21 days apart. Starting in the first week of December 2020, questions concerning COVID-19 vaccination were incorporated into the weekly questionnaire. In October 2021, during the second study phase, the Peruvian Ministry of Health recommended a heterologous booster dose using the Pfizer-BioNTech vaccine. Vaccination efforts in Peru consistently prioritized HCWs and high-risk populations [22, 23].

Vaccination status was categorized as follows: unvaccinated (no doses received), partially vaccinated (a single dose or second dose within 14 days prior to specimen collection), fully vaccinated with the primary vaccine series (second dose >14 days prior to specimen collection), and vaccinated with the primary series plus booster (receipt of 3 or more doses >14 days prior to specimen collection). Due to the simultaneous phase III placebo-controlled clinical trial of the BBIBP-CorV vaccine that began recruiting HCWs in Lima in early September 2020, participants involved in the trial or who completed follow-up before their vaccination status could be assessed were assigned an “unknown” vaccination status.

### SARS-CoV-2 Infection Detection

Self-collected nasal swabs were analyzed for SARS-CoV-2 using real-time reverse transcription polymerase chain reaction (rRT-PCR) at the US Naval Medical Research Unit SOUTH (NAMRU SOUTH) in Lima, in accordance with Centers for Disease Control and Prevention (CDC) testing protocols [24]. To optimize resource use, rRT-PCR testing was performed in pools of 5 specimens. If a pool tested positive, each of the 5 individual specimens within it was then tested separately.

### Data Management

Data collection and management for the influenza and the COVID-19 cohorts were conducted using Standard Data (Standard Co), a secure cross-platform that supports electronic data collection, automated data quality checks, cleaning, and visualization. Surveys were administered by study staff, who read questions aloud and recorded participant responses to ensure accuracy and consistency.

### Statistical Analysis

We assessed characteristics of HCWs potentially associated with testing positive for SARS-CoV-2 during follow-up. Categorical variables were summarized as frequencies and percentages, whereas continuous variables were expressed using medians and interquartile ranges. Differences in categorical variables were evaluated using Fisher exact test, and continuous variables were analyzed with the Wilcoxon rank-sum test. These tests were also used to compare participant characteristics and exposure frequencies across 2 study periods. Although some participants appeared in both periods, potentially affecting the assumption of independence in these tests, *P*-values were retained to provide descriptive context.

Associations between baseline characteristics, exposures, and SARS-CoV-2 positivity were assessed using generalized estimating equations (GEE) to account for the correlated structure of repeated measures and clustering by study site. Unless otherwise specified, all reported odds ratios should be interpreted as adjusted odds ratios, reflecting this clustering adjustment.

Infection occurrence was monitored through weekly self-collected nasal swabs tested for SARS-CoV-2 using real-time reverse transcription polymerase chain reaction (rRT-PCR). Person-time, or the time at risk, was calculated by counting the days between negative PCR results. A maximum cap of 10 days between tests was established, based on the observed performance of the test, indicating that an infection occurring within this timeframe would likely be detected by the subsequent test [25]. Days beyond this period were considered to represent unknown infection status and were excluded from both the analysis and the person-time calculation.

When a participant tested positive, indicating an incident infection, the period from the last negative test was split, allocating half the days to the at-risk period and the remainder as not at risk due to the infection. Aligned with similar studies, HCWs were deemed not at risk and were not assessed for subsequent infections for 60-days following a positive PCR result [26]. We assessed differences in time at risk using the Wilcoxon Mann–Whitney *U* test.

To explore the association between weekly reported exposures and PCR-confirmed SARS-CoV-2 positivity, we employed bivariate generalized estimating equations (GEE) models, incorporating study site to account for within-site correlation. These models used a binomial distribution with a logit link function and accounted for the correlated structure of participants’ repeated measures.

The percentage of follow-up weeks during which each exposure was reported was calculated across all participant-weeks to describe overall exposure frequency. Odds ratios were estimated to assess the association between reporting an exposure in a given week and testing positive for SARS-CoV-2 in that same week. These odds ratios were derived using GEE models, which account for the time-varying nature of both exposures and outcomes, as well as repeated measures within participants.

To analyze the predictive value of reported symptoms for COVID-19, we implemented conditional nonparametric regression through classification trees. This approach uses recursive partitioning, starting with the entire data set and progressively dividing it into subsets based on the values of input variables, creating child nodes. Each potential split was evaluated using χ^2^ tests, with splits selected to minimize *P*-values.

Statistical significance was defined as *P*-value ≤.05. Confidence intervals were reported to reflect estimate precision; intervals excluding 1.0 were interpreted as indicating statistical significance. All analyses were conducted using R software, version 4.1.3.

### Ethical Approval and Ethical Considerations

The study protocol was reviewed and approved by the institutional review boards (IRB) at NAMRU SOUTH and both participating study hospitals.^1^ Protocol and local IRB approvals were reviewed by the CDC's human subjects protection office and covered by the universal reliance agreement. All participants provided written informed consent prior to their involvement in the study.

(^1^See 45 C.F.R. part 46.101(c); 21 C.F.R. part 56.)

## RESULTS

### Participant Recruitment and Follow-up

In the first phase of the Peru Healthcare Worker COVID Cohort (PHCWCC-1), 760 HCWs were evaluated for eligibility. Of these, 667 (88%; 667/760) were eligible and consented to participate, completing both the baseline and at least 1 follow-up visit. At the Cayetano hospital site, 369 participants (54%; 369/667) were enrolled, compared to 298 (46%; 298/667) at the Loayza hospital site. There was no significant difference in participation rates between the 2 sites and complete follow-up, defined as 16 weeks of consistent completion of the questionnaire and respiratory sample self-collection, with 83% (308/369) completion at Cayetano and 88% at Loayza (263/298; *P* = .10).

In the subsequent phase (PHCWCC-2), 795 HCW were assessed for eligibility, with 702 (88%: 702/795) being eligible and completing both the baseline and at least 1 follow-up visit. Of these, 541 (77%; 541/702) had also participated in PHCWCC-1. The Cayetano site accounted for 383 participants (55%; 383/702), and the Loayza site for 319 (45%; 319/702), with no significant difference in participation rates between the sties. The complete follow-up rate for this phase was markedly lower at 54% (381/702), also with significant variability between sites: 77% at Cayetano (297/383) and 26% at Loayza (84/319; *P* < .001). The average number of follow-up visits per participant exceeded 15 visits in both phases but was significantly lower in the second phase compared to the first (Phase 1: 15.6 visits, Phase 2: 15.1 visits; *P* < .001).

Overall, participants in the second study phase were, on average, one year older, with the median age increasing from 44 to 45 years (*P* = .01). Additionally, a significantly higher proportion of participants were categorized under “other clinical” roles, which include patient care positions other than nurses and physicians (*P* < .02). No significant differences were observed between cohort participants across the 2 phases concerning assigned work department (*P* = 1.0), work in multiple facilities (*P* = .6), or self-reported gender (*P* = 1.0) as detailed in Table 1.

**Table 1.** Participant Characteristics and Self-reported Exposure and Safety Assessment by Follow-up Period: Peru Healthcare Worker COVID Cohort Phase 1 (PHCWCC-1: August 2020 to May 2021) and Phase 2 (PHCWCC-2: August 2021 to May 2022)

	PHCWCC-1 N (%)	PHCWCC-2 N (%)	*P*-value
**Study site**	667 (100)	702 (100)	.8
Cayetano	369 (55%)	383 (55%)	
Loayza	298 (45%)	319 (45%)	
**SARS-CoV-2 diagnostic test results throughout follow-up.**	667	702	.01
Positive (One or more)	75 (11%)	114 (16%)	
Negative (All)	592 (89%)	588 (84%)	
**Median age in year (IQR)**	44 (37, 51)	45 (38, 53)	.01
**Sex**	667	702	>.99
Female	411 (76%)	537 (76%)	
Male	128 (24%)	165 (24%)	
**How would you describe your current occupation?**	667	702	.02
Nurse	392 (59%)	402 (57%)	
Physician	45 (6.7%)	43 (6.1%)	
Other (clinical)	40 (6.0%)	75 (11%)	
Non-clinical	190 (28%)	182 (26%)	
**In which departments or wards are you primarily assigned to work?**	667	702	>.99
Emergency department	9 (1.3%)	8 (1.1%)	
Intensive care unit	41 (6.1%)	48 (6.8%)	
Inpatient (other)	587 (88%)	618 (88%)	
Outpatient	30 (4.5%)	28 (4.0%)	
**Currently working in more than 1 facility**	667	702	.68
Yes	102 (15%)	114 (16%)	
**Rate your risk of acquiring COVID-19 at the hospital, from 0 (no risk) to 10 (highest risk)**	667	702	<.001
Risk reported ≤8 (low to medium)	495 (74%)	459 (65%)	
Risk reported as 9 or 10 (high risk)	172 (26%)	243 (35%)	
**Would you provide direct care to a suspected COVID-19 patient at your facility?**	651	680	<.001
Yes	502 (77%)	574 (84%)	
**Have you been exposed to a suspected or confirmed COVID-19 case, excluding known negatives?**	650	656	<.001
Yes	413 (64%)	253 (39%)	
**In the past 14 d, did you provide direct care to a suspected or confirmed COVID-19 patient?**	656	698	.02
Yes	581 (89%)	587 (84%)	
**In the last 2 wks, have you had close contact with a colleague confirmed to have had COVID-19?**	633	669	<.001
Yes	309 (49%)	58 (8.7%)	
**During the last 2 wks, were you able to follow safety/PPE protocols while caring for COVID-19 patients?**	412	253	.26
Mostly	390 (95%)	245 (97%)	
Occasionally	22 (5.3%)	8 (3.2%)	
**Do you feel that you and your colleagues are safe from COVID-19 at work?**	663	698	<.001
No	616 (93%)	585 (84%)	

*P*-values reflect comparisons between the 2 periods. Although some participants were included in both periods, which may impact the assumption of independence, *P*-values are provided for descriptive purposes. All associations reflect adjustment for clustering by study site.

Abbreviations: COVID-19, coronavirus disease 2019; IQR, interquartile range; PPE, personal protective equipment; SARS-CoV-2, severe acute respiratory syndrome coronavirus 2.

### Contextual Timeline of Study Phases

**Figure 1.**
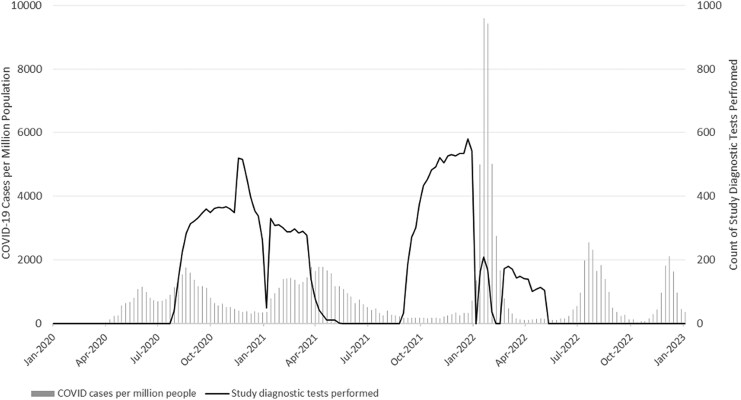
COVID-19 cases per million people and Peru Healthcare Worker COVID Cohort (PHCWCC) study diagnostic testing highlighting study follow-up relative to defined waves of SARS-CoV-2 infection in Peru, 2020–2022. Abbreviations: COVID-19, coronavirus disease 2019; SARS-CoV-2, severe acute respiratory syndrome coronavirus 2.

The cohort follow-up periods aligned with key phases of the COVID-19 pandemic in Peru. The first and second follow-up periods corresponded with the first and second waves of COVID-19 (PHCWCC-1), and the third wave (PHCWCC-2). These periods also coincided with significant events such as the COVID-19 vaccination rollout in February 2021 and the emergence of the Omicron variant in January 2022. The temporary drop in study diagnostics testing during June–July 2021 reflects a planned pause in study activities to align timelines with the parent study, Estudio Vacuna de Influenza Peru (VIP) (Figure 1).

### Vaccination

When COVID-19 vaccinations become available to HCWs in January 2021, 290 (39.1%; 290/664) participants were still within their follow-up period. The initial uptake of vaccine was high, with 66% (192/290) receiving their first dose within the first week and 90% (261/290) within the first 2 weeks of the vaccine rollout.

By the start of the second study phase in August 2021, 97% (680/702) of participants had received at least their primary 2-dose vaccine series. By the end of follow-up in May 2022, 6 months after booster doses were introduced in Peru [27], 75% (501/702) of participants had received a booster vaccination dose.

### Perception of Risk

During the first study phase (PHCWCC-1), 26% of participants reported high perceived risk of acquiring COVID-19 at the hospital (score ≥9), and 93% indicated that they felt unsafe in their work environment. In contrast, during the second study phase (PHCWCC-2), the proportion of participants reporting high perceived risk increased to 35% (*P* < .001), whereas those expressing that they felt unsafe at work decreased to 84% (*P* < .001).

These shifts in risk perception occurred in the context of significantly lower reported exposure to COVID-19 cases throughout the second study phase, including fewer recent exposures to COVID-19 patients (89% vs 84%, *P* = .02) and fewer close contacts with colleagues confirmed to have COVID-19 (49% vs 8.7%, *P* < .001).

During the first study phase, no significant differences were observed in the self-perceived level of COVID-19 infection risk between vaccinated and unvaccinated individuals (*P* = .41). Additionally, within the vaccinated group, perceptions of risk did not change significantly before and after vaccination (*P* = .67). However, due to near-universal vaccination during the second study phase, similar comparisons could not be performed.

### PCR Positivity During Follow-up

During the initial 16-week follow-up period of PHCWCC-1 (August 2020–May 2021), 75 HCWs tested positive for COVID-19, resulting in an infection rate of 1.15 per 1000 HCW days (95% confidence interval [CI]: .90, 1.14). In the subsequent follow-up period of PHCWCC-2 (August 2021–January 2022), 114 HCWs tested positive for SARS-CoV-2 via PCR, corresponding to an infection rate of 1.71 per 1000 HCW days (95% CI: 1.41, 2.05). The difference in incidence rates between the 2 periods was statistically significant, with an increase of 0.56 per 1000 HCW days (95% CI: .15, .97; *P* = .01). This represents approximately one additional infection for every 2000 HCW days during the PHCWCC-2 follow-up compared to the PHCWCC-1 period.

### Reported Symptoms

Throughout PHCWCC-1, 52% of the 75 participants who tested positive reported experiencing at least one symptom consistent with COVID-19. This proportion increased to nearly 80% during the second phase (PHCWW-2). Notably, the symptoms reported in PHCWCC-2 tended to be more prevalent yet milder compared to those in PHCWCC-1. Significant differences included an increase in the prevalence of cough (31% in PHCWCC-1 vs 52% in PHCWCC-2; *P* = .006), runny nose (25% vs 57%; *P* = .004), sore throat (21% vs 60%; *P* < .001), and muscle aches (20% vs 39%; *P* = .007). Conversely, changes in sensory perceptions such as taste (19% vs 8%; *P* = .05) and smell (19% vs 5%; *P* = .008) were more frequently reported PHCWCC-1. The incidence of fever (22%; *P* = .8) and shortness of breath (10%; *P* = .5) showed no significant differences between the follow-up periods. No infections required hospitalization or resulted in death.

**Figure 2.**
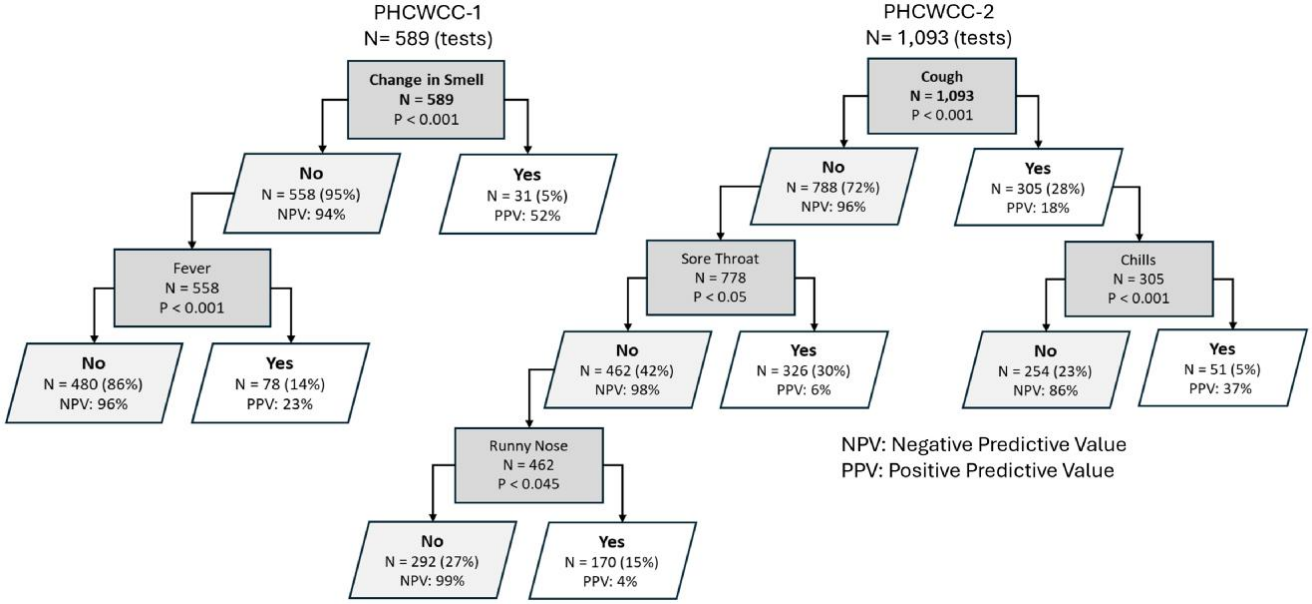
Conditional nonparametric regression classification trees for predicting SARS-CoV-2 PCR positivity from reported symptoms across follow-up periods: Peru Healthcare Worker COVID Cohort Phase 1 (PHCWCC-1: August 2020 to May 2021) and Phase 2 (PHCWCC-2: August 2021 to May 2022). Abbreviations: NPV, negative predictive value; PCR, polymerase chain reaction; PPV, positive predictive value; SARS-CoV-2, severe acute respiratory syndrome coronavirus 2.

Further analysis revealed that the predictive value of reported symptoms also varied by follow-up period. During PHCWCC-1, changes in smell and fever had significant predictive values of COVID-19 (both *P* < .001). In contrast, PHCWCC-2 identified cough, chills, sore throat, and runny nose as symptoms with significant predictive value, although their predictive strength was lower compared to the first period (Figure 2).

### Risk Factors for PCR Positivity During Follow-up

After accounting for variations between study facilities through clustering adjustment, several baseline characteristics were associated with decreased odds of testing positive for SARS-CoV-2 during follow-up of the first study phase. Being male, working in more than 1 facility, and being a nurse compared to non-clinical staff each was significantly associated with reduced likelihood of testing positive (*P* < .01). Clinical staff who reported that they would expect to provide direct care to suspected COVID-19 patients, based on their role, were also less likely to test positive (*P* < .01).

In the second phase, similar to the first, working in multiple facilities continued to be associated with a decreased likelihood of testing positive (*P* < .01). HCWs in the intensive care unit, compared to those working in the emergency department, were less likely to test positive (*P* = .03). HCWs with experience in caring for COVID-19 patients who reported a reduced ability to follow safety and PPE protocols were more likely to test positive for SARS-CoV-2 during PHCWCC-2 (*P* < .01).

Across both study phases, the perception of risk was found to significantly influence SARS-CoV-2 positivity, with HCWs who perceived a higher overall risk of infection being at a significantly lower risk of testing positive (*P* < .01). Findings are detailed in Table 2.

**Table 2.** Association Between Characteristics and Reported Exposures at Enrollment and Testing SARS-CoV-2 Positive During Follow-up: Peru Healthcare Worker COVID Cohort Phase 1 (PHCWCC-1: August 2020–May 2021) and Phase 2 (PHCWCC-2: August 2021–May 2022)

	PHCWCC-1			PHCWCC-2		
	OR	95% CI	*P*-value	OR	95% CI	*P*-value
**Sex**						
Female	**Reference**	…	…	**Reference**	…	…
Male	0.86	0.76–0.96	<.01	0.85	0.57–1.25	.40
**Current occupation**						
Non-clinical	**Reference**	…	…	**Reference**	…	…
Nurse	0.47	0.46–0.49	<.01	0.68	0.22–2.06	.49
Nurse assistant	0.98	0.78–1.25	.90	0.80	0.44–1.44	.46
Other	0.70	0.40–1.21	.20	0.83	0.74–1.85	.65
Physician	0.45	0.05–3.98	.47	0.54	0.18–1.60	.27
**Department or ward primarily assigned**						
Emergency department	**Reference**	…	…	**Reference**	…	…
Intensive care unit	1.03	0.93–1.14	.60	0.67	0.47–0.97	.03
Inpatient (other)	1.26	0.54–2.92	.59	0.86	0.60–1.23	.42
Outpatient	1.46	0.99–2.16	.06	2.23	0.44–11.3	.33
**Currently working in more than 1 facility**						
Yes, works in ≥2 facilities	0.73	0.70–0.76	<.01	0.56	0.38–0.81	<.01
**Rate your risk of acquiring COVID-19 at the hospital, from 0 (no risk) to 10 (highest risk)**						
Risk reported ≤8 (low to medium)	**Reference**	…	…	**Reference**	…	…
Risk reported as 9 or 10 (high risk)	0.94	0.93–0.95	<.01	0.81	0.78–0.85	<.01
**Would you provide direct care to a suspected COVID-19 patient at your facility?**						
Yes, would expect to provide direct care	0.65	0.60–0.71	<.01	0.61	0.34–1.12	.11
**Any exposure to a suspected or confirmed COVID-19 case**						
Yes, reported exposure	0.91	0.77–1.07	.27	0.89	0.51–1.56	.70
**Direct contact with any patient suspected of COVID-19?**						
Yes, reported in last 2 weeks	0.79	0.29–0.21	.64	1.30	0.63–2.64	.47
**Close contact (within 2 meters) with a colleague confirmed to have had COVID-19?**						
Yes, reported in last 2 weeks	1.05	0.92–1.21	.44	0.96	0.81–1.14	.65
**Able to follow safety/PPE protocols while providing care**						
Reports: “Always” or “most of the time”	**Reference**	…	…	**Reference**	…	…
Reports: “Occasionally” or “Rarely”	0.80	0.50–1.28	.36	1.87	1.62–2.17	<.01
**Do you feel that you and your colleagues are safe from COVID-19 at work?**						
Yes, reports feeling safe from COVID-19	0.72	0.24–2.20	.57	1.04	0.74–1.45	.81

Abbreviations: CI, confidence interval; COVID-19, coronavirus disease 2019; OR, odds ratio; PPE, personal protective equipment; SARS-CoV-2, severe acute respiratory syndrome coronavirus 2.

### Weekly Exposures and Incident SARS-CoV-2 Positivity

We also examined the impact of five types of self-reported weekly exposures on SARS-CoV-2 positivity within the same week:

- Exposure to a suspected or confirmed COVID-19 case.
- Direct contact with any patient suspected of having COVID-19.
- Close contact (within 2 meters) with a colleague confirmed to have had COVID-19.
- Direct contact with environments where COVID-19 patients were cared for.
- Situations where N95 respirators were unavailable.

During PHCWCC-1, the frequency of all reported exposures was notably higher compared to the subsequent phase (PHCWCC-2). Specifically, exposure to a COVID-19 case, direct contact with COVID-19 patients, and direct contact with environments where COVID-19 patients were treated were each reported in about 50% of weekly reports during PHCWCC-1. In contrast, these exposures were reported in only about 20% of weekly reports during PHCWCC-2. Similarly, close contact with colleagues confirmed to have COVID-19 decreased from 18% to 6%, and report of unavailable N95 respirators reduced from 6% to 3% between the 2 phases.

In PHCWCC-1, HCW who reported close contact with a colleague confirmed to have COVID-19 within the last 7 days had greater odds of testing positive for SARS-CoV-2 (*P* < .001). In contrast, during PHCWCC-2, all examined exposures were significantly associated with SARS-CoV-2 positivity, including exposure to confirmed cases, direct contact with suspected patients, close contact with infected colleagues, environments where patients were treated, and unavailability of N95 respirators (*P* < .001 for each) (Table 3).

**Table 3.** Prevalence of Reported Exposures and Their Association With SARS-CoV-2 Positivity Among Healthcare Workers in Lima, Peru, During PHCWCC-1 (August 2020 to May 2021) and PHCWCC-2 (August 2021 to May 2022) Follow-up Periods

	PHCWCC-1			PHCWCC-2		
Exposure Reported in the Prior 7 days	Reported*	OR	*P*-value	Reported*	OR	*P*-value
Any exposure to a suspected or confirmed COVID-19 case	56%	1.31	.27	25%	7.33	<.001
Direct contact with any patient suspected of COVID-19?	47%	0.96	.85	18%	3.91	<.001
Close contact (within 2 meters) with a colleague confirmed to have had COVID-19?	17%	2.37	<.001	6.9%	13.8	<.001
Direct contact with the environment where a COVID-19 patient was cared for	50%	0.83	.42	20%	3.67	<.0001
N95 Respirators not available	5.9%	0.85	.79	3%	3.13	<0.001

Abbreviations: COVID-19, coronavirus disease 2019; OR, odds ratio; PHCWCC, Peru Healthcare Worker COVID-19 Cohort.

^*^Percentages represent the proportion of follow-up weeks during which each exposure was reported, regardless of polymerase chain reaction (PCR) result. Odds ratios were estimated using generalized estimating equations (GEE) to assess the association between reporting the exposure in a given week and testing positive for severe acute respiratory syndrome coronavirus 2 (SARS-CoV-2) that same week, accounting for repeated measures and facility-level variation.

Additionally, we assessed HCW engagement in 15 aerosolizing procedures as defined by the WHO. Reports of exposure to these procedures remained consistent across both follow-up periods, with about 18% of weekly reports indicating engagement. No significant differences were observed between study phase, nor was there an association with SARS-CoV-2 positivity (Supplementary Table).

## DISCUSSION

During the early stages of the COVID-19 pandemic, Peru encountered significant challenges, including the highest case mortality rate in South America and concern about the protection of its healthcare system and workforce. In this context, our study revealed that despite substantial exposure to COVID-19—with over 84% of HCWs reporting direct contact with suspected cases shortly before enrollment—participants reported access to and a strong understanding of the necessary protective equipment and safety protocols. Despite these provisions, nearly one-third felt that their risk of acquiring COVID-19 at the hospital to be high, and over 90% of HCWs did not feel safe from infection at work.

An important observation from the study was the inverse association between perceived risk and incident infection. HCWs who perceived a higher risk of infection consistently exhibited lower rates of SARS-CoV-2 positivity. This suggests that a greater awareness of risk may lead to more diligent adherence to protective measures, potentially reducing the actual risk of infection. This pattern, where perceived risk of infection positively influences preventative behavior, has been observed in other health contexts as well [28].

These findings suggest that institutions could strengthen infection prevention by incorporating risk perception training into existing programs. Regular assessments of perceived risk could help identify groups with lower risk awareness, allowing for tailored education campaigns and safety protocols that address specific gaps. Emphasizing how individual behaviors impact personal and patient safety, using real-world examples, and providing regular feedback on infection trends could further reinforce risk awareness and promote consistent adherence to protocols.

Vaccination rates in the study cohorts were high, with 97% having received their primary vaccine series, and 75% reported a booster dose by the end of the second phase. Despite this, becoming vaccinated was not significantly associated with a decreased perception of the risk of becoming infected among the cohort. The reason for this finding is uncertain. It is possible that it reflects an understanding of the partial protection offered by vaccines, along with appreciation of the ongoing relevance of breakthrough infections among HCWs.

The frequency of reported occupational exposures notably declined between study phases. This reduction may reflect improved role differentiation and task allocation within the healthcare workforce or changing recognition of such exposures. Concurrently, specific work-related activities—such as direct contact with COVID-19 cases, close contact with infected colleagues, or interactions in patient care environments—were identified as significant predictors of SARS-CoV-2 infection detected within the same week of these exposures during the second phase (see Table 3). These findings suggest that the risk of infection was closely linked to the timing of the exposure, highlighting an increased risk of infection shortly after such contacts, even as overall reports of exposures declined. Additionally, the increased prevalence of more transmissible SARS-CoV-2 strains during the second phase, compared to earlier, less transmissible strains, may have contributed to differences observed between the two phases.

Notably, close contact with infected colleagues was the only significant risk factor common to both study phases. This consistent finding highlights the critical role of social interactions within healthcare settings in influencing infection risk among healthcare workers during both the early and middle phases of the pandemic. Less adherence to infection prevention guidelines in non-clinical shared spaces—such as nursing stations, break rooms, and dining halls—has been repeatedly identified as a key factor contributing to transmission among healthcare workers [29–31]. This pattern underscores the need for rigorous and continuous adherence to safety protocols, especially in communal areas where informal interactions can lead to lapses in protective measures.

During the second study phase, although infrequently reported, an unavailability of N95 respirators was identified as a significant risk factor for SARS-CoV-2 positivity. WHO recommendations at the time of the study and currently do not broadly distinguish between the use of respirators and medical masks for HCW protection against SARS-CoV-2 infection, except when specifying the use of respirators over medical masks primarily during aerosol-generating procedures for patients with COVID-19 [32]. Although our findings do not provide any evaluation of this guidance, they do highlight a role for consistent availability of personal protective equipment (PPE), including N95 respirators, in preventing the spread of infection among well-trained HCWs in healthcare settings.

Throughout both follow-up periods, the frequency of reported aerosol generating procedures (AGPs) were similar, and no association was observed with SARS-CoV-2 positivity. Although there has been debate about the extent of increased infection risk to healthcare workers from AGPs and what procedures fall under this category, both the CDC and WHO acknowledge that AGPs may increase the risk of infection and require enhanced infection prevention measures [32, 33]. Throughout both follow-up periods, the frequency of reported AGPs was similar and no association was observed with SARS-CoV-2 positivity. This suggests that HCWs were generally following recommended infection prevention protocols and that necessary equipment were available. Although there has been debate about the extent of increased infection risk to HCWs from aerosol-generating procedures, both the CDC and WHO acknowledge these procedures as requiring enhanced infection prevention measures [30, 31, 32, 33]. Our findings provide limited real-world observation suggesting that the risk associated with such procedures can be managed in settings where staff have adequate training and consistent access to appropriate personal protective equipment.

Our observations regarding changes in COVID-19 symptoms are corroborated by findings from Whitaker et al in a large UK cohort study and meta-analysis [32, 33], indicating a decreased predictiveness of sensory changes such as alterations in taste and smell with the emergence of the Omicron variant, compared to earlier variants like Alpha and Delta [32, 33, 34]. These studies suggest that sensory changes were more indicative of infection during the initial phase of the pandemic but became less predictive as new variants emerged. Conversely, negative predictive values for symptom-based screening, as shown in Figure 2, were considerably higher than positive predictive values across both study phases, underscoring the utility of symptom absence in ruling out infection, even as predictive symptom patterns evolved. Influenza-like symptoms, including cough, runny nose, sore throat, and muscle aches, were found to be more indicative of the Omicron variant. Shifts in the presentation of key symptoms may be attributed to changes in the immunization status of the population and/or alterations in the cellular tropism of SARS-CoV-2 with the newer variants [34]. Regardless, findings highlight the dynamic nature of the virus and the need for continual updates to symptom-based screening protocols to reflect an evolving epidemiological landscape.

Our findings should be interpreted in light of several limitations. First, data collection at baseline and through weekly questionnaires relied on self-reported information, which can introduce biases such as social desirability and recall bias. However, given that this study involved an established and experienced HCW cohort with a long history of engagement, we expect these biases were minimized.

Second, the study was designed and implemented during periods of pandemic uncertainty and an active emergency response. These conditions necessitated some modification to data collection and schedules as well as adaptation to follow-up procedures to accommodate rapidly changing health protocols and workforce availability. Such changes may have led to inconsistencies in the data gathered across different phases and potentially affected the comparability of findings. To address this, we adjusted for key confounding variables, including study site and participant role, and stratified analyses by study phase, as the primary time period definition. Although these measures aimed to minimize bias, some residual confounding may remain.

Additionally, we did not collect information on household or community exposures, which likely contributed to infection risk and limits our ability to fully account for non-occupational sources of exposure in our analysis.

Among the key strengths of our study is the frequency and breadth of sampling, which allowed for a more comprehensive detection of SARS-CoV-2 infection than would be possible with passive surveillance systems alone. The use of weekly testing and surveys provided granular, time-sensitive data on both exposures and outcomes, enabling the identification of transient risk factors and short-term behavioral changes that passive surveillance would likely miss. Additionally, maintaining a substantial proportion of the same cohort across both follow-up periods provided a longitudinal perspective, capturing the evolution of risk factors during both the pre- and post-vaccination phases. This longitudinal design strengthened our ability to track shifts in infection dynamics, exposure patterns, and risk perceptions over time, offering deeper insights into healthcare worker vulnerabilities during different phases of the pandemic.

In conclusion, this study provides insights into the evolving risks and protective factors for SARS-CoV-2 infection among HCWs in Peru during 2 distinct phases of the pandemic. The findings emphasize the importance of cohort studies to identify risk factors to inform mitigation measures. The relationship between perceived risk and infection rates highlights the role of risk awareness in encouraging protective behaviors and underscores the importance of education on infection risk, access to personal protective equipment, and adherence to safety protocols—even when not performing clinical activities—during periods of high community transmission.

These findings suggest that healthcare administrators and policymakers should consider prioritizing the implementation of enhanced workplace safety protocols, incorporating regular risk perception assessments into infection prevention programs, and ensuring the continuous availability of essential resources such as PPE. Additionally, addressing psychological stressors among HCWs through mental health support initiatives and fostering transparent communication about risks and safety measures can further strengthen protective behaviors.

These insights offer key considerations for future public health strategies aimed at protecting HCWs and maintaining the resilience of healthcare systems during infectious disease outbreaks.

## Supplementary Material

ciaf343_Supplementary_Data

## Supplementary Data

Supplementary materials are available at *Clinical Infectious Diseases* online. Consisting of data provided by the authors to benefit the reader, the posted materials are not copyedited and are the sole responsibility of the authors, so questions or comments should be addressed to the corresponding author.

## Notes

***Acknowledgments.*** The authors thank the healthcare workers who participated in this study and the staff at Cayetano Heredia Hospital and Archbishop Loayza National Hospital for their support. They also acknowledge the research and laboratory teams at NAMRU SOUTH for their assistance with data collection and SARS-CoV-2 testing. Their appreciation extends to the Centers for Disease Control and Prevention (CDC) and the Peruvian Ministry of Health for their support, as well as the Peru Healthcare Worker COVID Cohort (PHCWCC) and Estudio Vacuna de Influenza Peru (VIP) teams for their contributions to the study.

***Copyright statement.*** Some authors are federal employees of the United States government. This work was prepared as part of our official duties. Title 17 U.S.C. 105 provides that “copyright protection under this title is not available for any work of the United States Government.” Title 17 U.S.C. 101 defines a US Government work as work prepared by a military service member or employee of the US Government as part of that person's official duties.

***Financial support.*** The study was funded through CDC Interagency Agreements: 16FED1612328, 16FED1612343, 18FED1812054IPD, and 19FED19112003IPD. The study protocol (NAMRU6 2019.0011) was approved by the NAMRU SOUTH Institutional Review Board in compliance with all applicable federal regulations governing the protection of human subjects.

## References

[1] World Health Organization . WHO calls for healthy, safe and decent working conditions for all health workers, amidst COVID-19 pandemic. 2020. Available at: https://www.who.int/news/item/28-04-2020-20200428_protect_workers. Accessed 1 May 2024.

[2] Black JRM, Bailey C, Przewrocka J, Dijkstra KK, Swanton C. COVID-19: the case for health-care worker screening to prevent hospital transmission. The Lancet 2020; 395:1418–20. 10.1016/S0140-6736(20)30917-X PMC7162624 32305073

[3] Rodrigues D, Savarese M. Brazil confirms first coronavirus case in Latin America. 2020. Available at: https://apnews.com/article/fd3d0d0120dd10f3d09bad78a4dd9539. Accessed 2 February 2024.

[4] Aquino M, Garrison C. Peru records first confirmed case of coronavirus, President Vizcarra says. 2020. Available at: https://www.reuters.com/article/us-health-coronavirus-peru/peru-records-first-confirmed-case-of-coronavirus-president-vizcarra-says-idUSKBN20T1S9/. Accessed 31 January 2024.

[5] Johns Hopkins Center for Systems Science and Engineering (CSSE) . Mortality Analysis. 2023. Available at: https://coronavirus.jhu.edu/data/mortality. Accessed 2 February 2024.

[6] Inga-Berrospi F, Arosquipa Rodríguez C. Avances en el desarrollo de los recursos humanos en salud en el Perú y su importancia en la calidad de atención. Rev Peru Med Exp Salud Pública 2019; 36:312. 31460646 10.17843/rpmesp.2019.362.4493

[7] Maguiña Vargas C. Reflexiones sobre el COVID-19, el Colegio Médico del Perú y la Salud Pública. ACTA MEDICA Peru 2020; 37:8–10. 10.35663/amp.2020.371. Available at: http://amp.cmp.org.pe/index.php/AMP/article/view/929.

[8] Neyra-León J, Huancahuari-Nuñez J, Díaz-Monge JC, Pinto JA. The impact of COVID-19 in the healthcare workforce in Peru. J Public Health Policy 2021; 42:182–4. 33028932 10.1057/s41271-020-00259-6 PMC7539549

[9] Yıldırım M, Arslan G, Özaslan A. Perceived risk and mental health problems among healthcare professionals during COVID-19 pandemic: exploring the mediating effects of resilience and coronavirus fear. Int J Ment Health Addict 2022; 20:1035–45. 33223977 10.1007/s11469-020-00424-8 PMC7668285

[10] Yin Q, Chen A, Song X, Deng G, Dong W. Risk perception and PTSD symptoms of medical staff combating against COVID-19: a PLS structural equation model. Front Psychiatry 2021; 12:607612. 33658951 10.3389/fpsyt.2021.607612 PMC7917132

[11] Lasalvia A, Amaddeo F, Porru S, et al Levels of burn-out among healthcare workers during the COVID-19 pandemic and their associated factors: a cross-sectional study in a tertiary hospital of a highly burdened area of north-east Italy. BMJ Open 2021; 11:e045127. 10.1136/bmjopen-2020-045127 PMC7813385 33455940

[12] Lam SC, Arora T, Grey I, et al Perceived risk and protection from infection and depressive symptoms among healthcare workers in mainland China and Hong Kong during COVID-19. Front Psychiatry 2020; 11:686. 32765321 10.3389/fpsyt.2020.00686 PMC7378321

[13] Puci MV, Nosari G, Loi F, Puci GV, Montomoli C, Ferraro OE. Risk perception and worries among health care workers in the COVID-19 pandemic: findings from an Italian survey. Healthc Basel Switz 2020; 8:535. 10.3390/healthcare8040535 PMC7761765 33287260

[14] Migisha R, Ario AR, Kwesiga B, et al Risk perception and psychological state of healthcare workers in referral hospitals during the early phase of the COVID-19 pandemic, Uganda. BMC Psychol 2021; 9:195. 34920763 10.1186/s40359-021-00706-3 PMC8678424

[15] Wise T, Zbozinek TD, Michelini G, Hagan CC, Mobbs D. Changes in risk perception and self-reported protective behaviour during the first week of the COVID-19 pandemic in the United States. R Soc Open Sci 2020; 7:200742. 33047037 10.1098/rsos.200742 PMC7540790

[16] Tomczyk S, Rahn M, Schmidt S. Social distancing and stigma: association between compliance with behavioral recommendations, risk perception, and stigmatizing attitudes during the COVID-19 outbreak. Front Psychol 2020; 11:1821. 32849073 10.3389/fpsyg.2020.01821 PMC7432118

[17] Roshanshad R, Roshanshad A, Molavi Vardanjani H,, et al Risk perception, attitude, and practice related to COVID-19: a cross-sectional study among 1085 Iranian healthcare workers. Ann Med Surg 2021; 70:102865. 10.1016/j.amsu.2021.102865 PMC8450226 34567550

[18] Niepel C, Kranz D, Borgonovi F, Emslander V, Greiff S. The coronavirus (COVID-19) fatality risk perception of US adult residents in March and April 2020. Br J Health Psychol 2020; 25:883–8. 32519364 10.1111/bjhp.12438 PMC7300951

[19] Thompson MG, Soto G, Peretz A, et al Influenza vaccine effectiveness within prospective cohorts of healthcare personnel in Israel and Peru 2016–2019. Vaccine 2021; 39:6956–67. 34509322 10.1016/j.vaccine.2021.07.077

[20] Porras AA. Empieza la vacunación en Perú mientras avanza la segunda ola de contagios. 2021. Available at: https://www.france24.com/es/am%C3%A9rica-latina/20210209-covid19hoy-noticias-coronavirus-vacunaci%C3%B3n-cuarentenas. Accessed 27 June 2024.

[21] Ministerio de Salud (MINSA) . ¿Quiénes serán vacunados? 2021. Available at: https://web.archive.org/web/20210223083717/Available at: https://www.minsa.gob.pe/vacuna-covid-19/? op=3. Accessed 27 June 2024.

[22] National Center for Immunization and Respiratory Diseases (U.S.) . Division of Viral Diseases. 2019-novel coronavirus (2019-nCoV) real-time rRT-PCR panel primers and probes. 2020; Available at: https://stacks.cdc.gov/view/cdc/84525. Accessed 4 February 2024.

[23] Mallett S, Allen AJ, Graziadio S, et al At what times during infection is SARS-CoV-2 detectable and no longer detectable using RT-PCR-based tests? A systematic review of individual participant data. BMC Med 2020; 18:346. 33143712 10.1186/s12916-020-01810-8 PMC7609379

[24] Hadley E, Yoo YJ, Patel S, et al Insights from an N3C RECOVER EHR-based cohort study characterizing SARS-CoV-2 reinfections and long COVID. Commun Med 2024; 4:129. 38992084 10.1038/s43856-024-00539-2 PMC11239932

[25] Bendezu-Quispe G, Caira-Chuquineyra B, Fernandez-Guzman D, Urrunaga-Pastor D, Herrera-Añazco P, Benites-Zapata VA. Factors associated with not receiving a booster dose of COVID-19 vaccine in Peru. Vaccines 2022; 10:1183. 35893832 10.3390/vaccines10081183 PMC9330573

[26] Houghton C, Meskell P, Delaney H, et al Barriers and facilitators to healthcare workers’ adherence with infection prevention and control (IPC) guidelines for respiratory infectious diseases: a rapid qualitative evidence synthesis. Cochrane Database Syst Rev 2020; 4:CD013582. 32315451 10.1002/14651858.CD013582 PMC7173761

[27] Zabarsky TF, Bhullar D, Silva SY, et al What are the sources of exposure in healthcare personnel with coronavirus disease 2019 infection? Am J Infect Control 2021; 49:392–5. 32795495 10.1016/j.ajic.2020.08.004 PMC7419261

[28] Mohan Y, Charumathi B, Anantha Eashwar VM, Jain T, Abiramasundari VK. Incidence and source of COVID-19 infection among health care workers in a tertiary hospital in south India: a prospective cohort study. Int J Prev Med 2022; 13:108. 36247191 10.4103/ijpvm.IJPVM_687_20 PMC9564233

[29] Mathabire Rücker SC, Gustavsson C, Rücker F, Lindblom A, Hårdstedt M. Transmission of COVID-19 among healthcare workers—an epidemiological study during the first phase of the pandemic in Sweden. Epidemiol Infect 2022; 150:1–36. 10.1017/S0950268822000231 PMC8987659 35272735

[30] US Centers for Disease Control and Prevention . Infection Control Guidance: SARS-CoV-2. 2024. Available at: https://www.cdc.gov/covid/hcp/infection-control/index.html. Accessed 26 September 2024.

[31] World Health Organization . Infection prevention and control in the context of COVID-19: A guideline. 2023. Available at: https://www.who.int/publications/i/item/WHO-2019-nCoV-IPC-guideline-2023.4. Accessed 26 September 2024.

[32] Esmaeili M, Abdi F, Shafiee G, et al Olfactory and gustatory dysfunction in 2019 novel coronavirus: an updated systematic review and meta-analysis. Int J Prev Med 2021; 12:170. 35070203 10.4103/ijpvm.IJPVM_484_20 PMC8724794

[33] Whitaker M, Elliott J, Bodinier B, et al Variant-specific symptoms of COVID-19 in a study of 1,542,510 adults in England. Nat Commun 2022; 13:6856. 36369151 10.1038/s41467-022-34244-2 PMC9651890

[34] Hui KPY, Ho JCW, Cheung M, et al SARS-CoV-2 omicron variant replication in human bronchus and lung ex vivo. Nature 2022; 603:715–20. 35104836 10.1038/s41586-022-04479-6

