# Supplementary material for: COVID-19 Exposure, Protective Measures, Symptom Assessment, and Risk Perception Among Healthcare Workers in Peru: A Longitudinal Cohort Study (2020–2021)
Source: Clin Infect Dis. 2025 Jul 29;81(3):467–77. doi: 10.1093/cid/ciaf343 (PMC12497964; doi:10.1093/cid/ciaf343)
Supplement: ciaf343_Supplementary_Data [file ciaf343_supplementary_data.docx]

# Supplemental Table

Supplemental Table: Frequency of Reported Aerosolizing Procedures and Association with SARS-CoV-2 Positivity Among Healthcare Workers in Lima, Peru During Follow-up PHCWCC-1 (August 2020 – May 2021) and PHCWCC-2 (August 2021 – May 2022)

|  | PHCWCC-1 | | | PHCWCC-2 | | |
| --- | --- | --- | --- | --- | --- | --- |
|  | Reported* | OR† | p-value | Reported* | OR† | p-value |
| Airway suctioning (Open) | 10% | 0.75 | 0.59 | 12% | 0.53 | 0.41 |
| Airway suctioning (Close) | 7.6% | 0.29 | 0.12 | 7.0% | 1.93 | 0.42 |
| Positive pressure: BiPAP, CPAP | 3.7% | 0.40 | 0.40 | 2.4% | -- | -- |
| High flow 02 | 5.6% | 0.41 | 0.25 | 3.4% | 1.02 | 0.98 |
| Manual (bag) ventilation | 3.3% | -- | -- | 3.6% | -- | -- |
| Nebulizer treatments | 1.3% | 1.25 | 0.85 | 1.7% | 2.72 | 0.31 |
| Intubation | 5.2% | .86 | 0.83 | 5.4% | -- | -- |
| Extubation | 3.9% | 1.14 | 0.86 | 3.8% | -- | -- |
| Code / CPR | 0.6% | -- | -- | 0.7% | -- | -- |
| Chest physiotherapy | 2.3% | 1.16 | 0.86 | 1.8% | 1.0 | 1.0 |
| Bronchoalveolar lavage | 0.3% | -- | -- | 0.3% | 9.62 | 0.12 |
| Breaking ventilation circuit | 0.7% | -- | -- | 0.5% | 7.1 | 0.27 |
| Sputum induction | 1.3% | -- | -- | 1.6% | 2.98 | 0.24 |
| Bronchoscopy | 0.1% | -- | -- | 0.2% | -- | -- |
| Any procedure (any of above) | 18% | 1.38 | 0.42 | 19% | 2.53 | 0.17 |

PHCWCC: Peru Healthcare Worker COVID-19 Cohort

* For all participants, the proportion of follow-up weeks that that the exposure was reported

† Odds Ratios were estimated using generalized estimating equations (GEE) to account for repeated measurements on patients and to control for variations across facilities.
